# Advancements in the treatment of mycosis fungoides and Sézary syndrome: monoclonal antibodies, immunotherapies, and Janus kinase inhibitors

**DOI:** 10.3389/fimmu.2023.1291259

**Published:** 2023-11-03

**Authors:** Iman Quadri, John C. Reneau, Walter Hanel, Catherine G. Chung

**Affiliations:** ^1^ The Ohio State University College of Medicine, Columbus, OH, United States; ^2^ Division of Hematology, The Ohio State University Wexner Medical Center and The James and Solove Research Center and Cancer Hospital, Columbus, OH, United States; ^3^ Department of Pathology, The Ohio State University Wexner Medical Center, Columbus, OH, United States; ^4^ Department of Dermatology, The Ohio State University Wexner Medical Center, Columbus, OH, United States

**Keywords:** cutaneous T-cell lymphoma, mycosis fungoides, Sézary syndrome, immunotherapy, immune checkpoint inhibitors, cancer therapeutics, monoclonal antibodies, Janus kinus inhibitors

## Abstract

Mycosis fungoides (MF) and Sézary syndrome (SS) are forms of cutaneous T cell lymphoma (CTCL) that pose significant challenges in their clinical management, particularly in refractory and advanced-stage disease. With the emergence of novel therapeutic modalities however, there are increasing opportunities to exploit the current understanding of pathophysiologic mechanisms of MF/SS for treatment. This review summarizes recent advances in the treatment of MF/SS, with a focus on monoclonal antibodies, immunotherapies, and Janus kinase (JAK) inhibitors, including ongoing clinical trials.

## Introduction

Cutaneous T-cell lymphoma (CTCL) is a group of non-Hodgkin’s lymphomas characterized by infiltration of the skin by malignant T lymphocytes. Mycosis fungoides (MF) is the most common subtype of CTCL and is usually characterized by skin-limited patches and plaques in sun-protected sites with indolent behavior. In advanced disease, there can be involvement of lymph nodes, blood, and/or visceral organs. Sézary syndrome (SS) is an aggressive form of CTCL that typically presents with erythroderma (erythema and scale affecting >80% of body surface area), lymphadenopathy, and leukemic blood involvement (1–3). The exact pathogenesis of MF/SS is not known, but several molecular and immunologic elements have been implicated in their progression, presenting potential targets for therapy. We discuss how recent advances in the understanding of the pathophysiologic mechanisms of CTCL allow for increased applications of current and novel treatments, including monoclonal antibodies, immune checkpoint inhibitors, and Janus kinus pathway inhibitors in advanced MF/SS.

## Monoclonal antibodies

Monoclonal antibodies are immunoglobulins that target epitopes or antigens and are utilized as immunomodulatory and cytokine-targeting agents for conditions including rheumatoid arthritis, multiple sclerosis, and psoriasis (4). In CTCL, CD52, C-C chemokine receptor 4 (CCR4), CD30, and CD25 have been exploited as therapeutic targets (5).

### Alemtuzumab

Alemtuzumab is an anti-CD52 monoclonal antibody that was initially approved by the FDA in 2001 for B-cell chronic lymphocytic leukemia (B-CLL) (6). CD52 is a small glycopeptide expressed on the cell surface of normal and malignant T lymphocytes, along with other leukocytes (7).Elevated CD52 expression on CD4^+^ T lymphocytes, the main circulating cell in SS, makes this molecule an applicable target (8). Alemtuzumab is historically considered the first monoclonal antibody treatment for MF/SS. It first showed promise as a CTCL therapy when a 2002 report showed an overall response rate (ORR) of 100% in 3 cases of CTCL with response duration of up to four years (5, 9).

Alemtuzumab underwent a phase II clinical trial in 2003 as a potential treatment in advanced MF/SS. The safety and efficacy of the drug was evaluated in 22 patients with advanced MF/SS who failed to respond to standard treatments of psoralen + ultraviolet A phototherapy (PUVA), radiotherapy, or chemotherapy. ORR to alemtuzumab was determined by administering the drug using an escalating dose regimen of 3 mg, 10 mg, and finally target dose of 30 mg, three times weekly for up to 12 weeks (10). The ORR was 55% in the 22 MF/SS patients, with 32% reaching complete remission and 23% having partial remission. The ORR in SS patients (n=7) was 86%. Adverse side effects to treatment included cytomegalovirus reactivation in four patients and suspected or confirmed infections in six patients (3 fever of unknown origin, 1 herpes simplex, 1 fatal aspergillosis, 1 mycobacterium pneumonia.) (10) Despite this, it was determined that alemtuzumab was a potentially safe and viable therapy for advanced MF/SS with the use of antibiotic and antiviral prophylaxis (10). In 2014, a retrospective analysis of 39 patients with advanced CTCL evaluated the long-term safety and efficacy of alemtuzumab treatment for MF/SS. Overall ORR was 51%; however, 62% of patients had grade 3 or higher infections and 26% had hematologic toxicity. For patients with SS, there was an ORR of 70% while in patients with MF, the ORR was only 25%, supporting alemtuzumab as a better treatment option for SS than MF (11). Since then, newer monoclonal antibodies with more favorable side effect profiles have been approved and/or have undergone clinical studies for treatment of MF/SS.

### Denileukin diftitox

Denileukin Diftitox (Dd) is a fusion protein combining diphtheria toxin and interleukin-2 (IL-2). Binding of Dd to IL-2 receptors (IL-2R) leads to internalization of the toxin and subsequent cell death (12). Dd was approved for the treatment of relapsed/refractory CTCL patients expressing CD25, the high-affinity subunit of IL-2R, under the brand name Ontak in 1999 (13, 14). In a phase III trial that evaluated differing doses of Dd (9 mcg/kg vs 18 mcg/kg) vs placebo, 144 patients with refractory CD25-positive stage 1A-3 MF or SS were enrolled. Patients were randomly assigned to Dd 9 mcg/kg (n=45), Dd 18mcg/kg (n=55), or placebo infusions (n=44). An ORR of 44% was reported in all Dd treated patients vs 16% ORR in the placebo (15, 16). The ORR was higher in the Dd 18mcg/kg treated group (ORR 49.1%) than the Dd 9 mcg/kg group (ORR 37.8%) (16). After determining Dd was an effective treatment option for CD25-positive disease, Dd in the treatment of “low” CD25 expression (<20% CD25 cells by immunohistochemistry staining) disease was evaluated in a subsequent study. 36 patients with low CD25 expression were treated with Dd 18 mcg/kg daily. ORR was 30.6%, suggesting that degree of CD25 expression is not a strong predictor of Dd response in MF/SS (15). Ontak was voluntarily removed from the market due to manufacturing issues in 2014 (17). A new and improved Dd, E777, has been remanufactured. A phase 3 trial was completed in 2021 for E777 use in persistent and recurrent CTCL (NCT01871727). E777 was administered intravenously (IV) to 112 patients with recurrent or refractory MF/SS for 5 consecutive days every 21 days for up to 8 cycles. Preliminary data showed ORR 36.2% for E777 therapy, indicating a clinical benefit similar to Ontak for these patients (17). A biologic license application (BLA) for E777 has been submitted and is currently awaiting FDA approval for persistent or recurrent CTCL.

### Mogamulizumab

Mogamulizumab is a humanized IgG1k monoclonal antibody with a defucosylated Fc region that selectively binds to C-C chemokine receptor 4 (CCR4) (18–20). CCR4 is a molecule involved in cell trafficking of lymphocytes to the skin and is expressed on the surface of malignant T- cells, making it an appropriate therapeutic target for CTCL (20–22). Mogamulizumab became the first anti-CCR4 biologic approved in 2012 when Japan approved it for the treatment of relapsed or refractory CCR4+ adult T cell Leukemia-Lymphoma (ATLL) (18, 19, 23). Treatment of CTCL by mogamulizumab was approved by the United States in 2018 after the landmark MAVORIC trial (20). In this phase III randomized controlled trial, patients who had previously failed at least one systemic therapy for MF/SS were randomized to treatment by mogamulizumab or vorinostat, an histone deacetylase (HDAC) inhibitor used in standard therapy of CTCL (24). The study looked at 372 patients who were assigned either mogamulizumab (n=186) or vorinostat (n=186) treatment. The median progression free survival for the mogamulizumab treated group was significantly higher at 7.7 months, compared to 3.1 months for the vorinostat-treated group (20). ORR was overall higher in all Mogamulizumab treated groups compared to Vorinostat. SS patients who underwent mogamulizumab therapy had an ORR of 37% (2% for vorinostat) compared to MF patients who had an ORR of 21% (7% for vorinostat). Blood involvement elicited the greatest response to mogamulizumab with 68% ORR (19% for vorinostat), followed by skin ORR of 42% (16% in vorinostat), and 17% with lymph node involvement (20).

Currently, a large prospective multi-institutional study, “Real-World Observational Study of Poteligeo (PROSPER) in Adult Patients with Mycosis Fungoides (MF) and Sézary syndrome (SS)” (NCT05455931), is investigating longer term outcomes of mogamulizumab therapy. Additionally, a number of current clinical trials are underway evaluating mogamulizumab therapy in conjunction with other treatments.

A phase I/II trial “Mogamulizumab and extracorporeal photopheresis for the treatment of Sézary syndrome or mycosis fungoides” (NCT04676087) is currently in progress to evaluate the safety and efficacy of mogamulizumab with extracorporeal photopheresis (ECP) and to determine if these treatments work better in combination than if they were administered in a singular fashion. ECP has been used alone and in combination with both skin-directed and systemic treatments in the palliative management of CTCLs for several decades (25). It is a relatively safe apheresis procedure that involves photosensitizing patient leukocytes with 8-methoxypsoralen (8-MOP), followed by UV-A light exposure before returning the leukocytes to the patient (26). It was first approved by the FDA in 1988 for SS (25). Eligibility criteria include a confirmed diagnosis of MF/SS having failed no more than three standard therapies. The study protocol includes an induction, treatment, and maintenance phase, starting with IV infusion of Mogamulizumab with extracorporeal photopheresis given on the same day over a determined treatment schedule. The results of this clinical trial may suggest that established CTCL treatments used in conjunction with newer immunotherapies could be a promising approach for discovering new therapeutic regimens for MF/SS.

The current trial “Addition of an anti-cancer drug, Hu5F9-G4 (magrolimab), to the usual chemotherapy treatment (mogamulizumab) in T-Cell (a type of immune cell) lymphoma that has returned after treatment or does not respond to treatment” (NCT04541017) is a phase IB/II trial evaluating magrolimab and mogamulizumab therapy together for recurrent or treatment refractory CTCL. Magrolimab is an inhibitor of CD47, a surface protein that regulates immune mediated phagocytosis (27). CD47 serves as a ligand that prevents phagocytosis when engaged. Magrolimab may thus lead to a further enhancement of phagocytosis of moga-targeted CTCL cells (28). This trial will compare ORR of mogamulizumab and magrolimab combined treatment versus treatment with mogamulizumab alone.

Mogamulizumab is also under being further investigated in the clinical trial “Third-party natural killer (NK) cells and mogamulizumab for the treatment of relapsed or refractory cutaneous T-cell lymphomas or adult T-cell leukemia/lymphoma” (NCT04848064). NK cells are thought to be beneficial in the treatment of cancer for several reasons. Their innate killing ability, selective action on CTCL cells due to their frequent downregulation of MHC receptors, enhanced cytotoxic activity, and demonstrated synergy with other cancer therapies have made NK cells a promising cellular therapy for CTCL. In addition, their off-the-shelf capability allows treatment initiation without a manufacturing step as in chimeric antigen (CAR) T-cell products. In this study, IL-21 expanded donor NK cells from healthy individuals are given in combination with mogamulizumab. Binding of NK cells to mogmulizumab may allow further enhanced on target CTCL killing. The trial uses an escalating dose of NK cells. Patients are administered IV mogamulizumab according to a determined treatment schedule before receiving NK cell infusions every other week for a total of six infusions followed by standard of care mogamulizumab monotherapy.

### Brentuximab vedotin

Brentuximab vedotin is an anti-CD30 antibody that is conjugated to monomethyl auristatin E that was developed for the treatment of Hodgkin lymphoma (29, 30). CD30 is a receptor expressed on activated effector or memory T-helper cells which signals through the NF-kappa B pathway. When CD30 is expressed in MF, there is reduced disease-specific survival associated with a more aggressive clinical profile including large cell transformation on histology (30). In 2017, Brentuximab was approved by the FDA for treatment of CD30+ MF and cutaneous anaplastic large cell lymphoma (C-ALCL) in patients who had received prior therapy based on results of the ALCANZA trial (31). The final results of the ALCANZA trial were published in 2020. The trial compared use of brentuximab to either bexarotene or methotrexate. The ORR was significantly greater in brentuximab-treated patients versus bexarotene or methotrexate. Additionally, complete response was reached in five brentuximab patients and in zero patients with the other treatments. Peripheral neuropathy was the main adverse event. The results of the study overall showed that brentuximab could be a preferential therapy for treatment of MF when biopsy samples show composition of at least 10% CD30+ malignant cells (31). Several current clinical trials are currently underway for Brentuximab use in MF and SS. As brentuximab may cause a dose and duration dependent toxicities, most notably peripheral neuropathy, recent studies have focused more on dosing of brentuximab for optimal outcomes. A non-randomized interventional trial (NCT03587844) aims to study the efficacy of brentuximab at a lower dose than that of FDA approval.

## Immune checkpoint Inhibitors

Immune checkpoint inhibitors were first identified when researchers began to discover the intricate mechanisms by which cancer cells evade the immune system. Specific immune checkpoints such as cytotoxic T-lymphocyte-associated protein 4 (CTLA-4) and programmed cell death protein 1 (PD-1) were identified as critical regulatory pathways that inhibit the T-cell response to cancer cells. Cancer cells exploit these checkpoints to evade detection and destruction by the immune system. By blocking these immune checkpoints with antibody inhibitors, the immune system can have a prolonged and robust response to various malignancies (32).

Ipilumumab, an anti-CTLA-4 antibody, was one of the pioneer immune checkpoint inhibitors studied and subsequently approved by the FDA in 2011 for treatment of metastatic melanoma (33). This then opened the door to explore PD-1 inhibitors which over time demonstrated success in treating several cancer types including melanoma and non-small cell lung cancer (NSCLC) (34). Over time, the role of the PD-1/PD-L1 axis in MF and SS has been more solidified, making immune checkpoint inhibitors, particularly PD-1 inhibitors pembrolizumab and nivolumab, a promising approach to therapy.

### Pembrolizumab

Pembrolizumab is a fully humanized IgG 4 kappa monoclonal antibody against PD-1 (34). This PD-1 inhibitor gained FDA approval in 2014 for specific indications of advanced melanoma pretreated by anti-CTLA 4 antibody, ipilumab (35, 36).

Results from a phase II trial of “Pembrolizumab in relapsed and refractory mycosis fungoides and Sézary syndrome” in 2020 showed an ORR of 38% in 24 heavily pre-treated MF/SS patients. There were 2 complete responses and 7 partial responses. There was worsening of erythroderma and pruritis in 53% of patients with SS that was transient and did not require treatment discontinuation. These flares correlated with high PD-1 expression on Sézary cells but not response to treatment. Of note, immune-related adverse side effects led to discontinuation of treatment in four patients (37).

A current phase II clinical trial is investigating pembrolizumab as an initial treatment for MF stages IB-IV (NCT03695471). Other clinical trials include pembrolizumab treatment in combination with gemcitabine for advanced MF and SS (NCT04960618) as well as with decitabine and/or pralatrexate in patients with relapsed or refractory PTCL or CTCL (NCT03240211).

### Nivolumab

Another PD-1 antibody of interest in CTCL treatment is nivolumab, although the literature is sparse in the use of Nivolumab for MF/SS. In a 2015 trial “Nivolumab in patients with relapsed or refractory hematologic malignancy,” 81 patients with heterogeneous T and B cell lymphomas were treated, including 13 MF patients. The objective response rate was 15% in MF patients (38). Nivolumab was combined with ipilumab for relapsed or refractory hematologic malignancies in a phase I study, including Hodgkin and non-Hodgkin B-cell lymphomas, multiple myeloma, and T cell lymphomas that included CTCLs and PTCLs. The ORR in CTCL was only 9% compared to 73% in the Hodgkin lymphoma group (39). A single case report of nivolumab for treatment of erythrodermic MF noted more than 12 months of disease control in a patient with advanced and heavily pretreated MF (40). A new trial evaluating the combination of nivolumab with duvelisib, a phosphoinositide 3- kinase (PI3K) inhibitor, for MF/SS (NCI-2020-11641). PI3K inhibitors are enzymes involved in cell signaling pathways that play a role in cancer cell growth and survival. Previous studies have shown that duvelisib has promising clinical activity in treating advanced hematologic malignancies, providing a potential therapeutic option (41). By inhibiting both tumor cell growth and immune checkpoints with duvelisib and nivolumab combined, a greater overall response may be seen with both inhibitors versus treatment with each alone.

## JAK-STAT inhibitors

Janus kinases (JAK) are a family of enzymes that are involved in the JAK-STAT (signal transducer and activator of transcription) signaling pathway. This pathway transmits signals from cytokines and growth factors that are essential for cellular functions such as immune responses, hematopoiesis, and inflammatory responses (42). By interfering with the JAK-STAT pathway through the use of small molecule inhibitors, the downstream effects of cytokines can be interrupted. JAK inhibitors have been a meaningful breakthrough as a treatment for dermatologic conditions including alopecia areata, atopic dermatitis, dermatomyositis, graft versus host disease (GVHD), psoriasis, and vitiligo (43–48). JAK inhibitors bind to the active site of Janus kinases and inhibit the enzymatic activity, blocking the phosphorylation of STAT proteins. These proteins normally enter the nucleus and regulate gene expression when activated. When this process is blocked, immune and inflammatory responses are suppressed (42). Although the pathophysiology of CTCLs is not completely understood, recent evidence has suggested a role of the JAK-STAT pathway in progression of CTCL. Specifically, somatic gene mutations of the JAK1 and JAK3 were shown to contribute to dysregulated JAK-STAT signaling in CTCLs (49–53). Inhibition of the JAK-STAT pathway could therefore be a novel target for MF/SS therapy (49).

### Ruxolitinib

Efficacy of JAK-STAT inhibitory therapy for T cell lymphomas was demonstrated in a phase II biomarker study of ruxolitinib in PTCL and MF patients (NCT02974647). The trial enrolled patients with T cell lymphomas, including 7 MF patients, with specific biomarkers that were assessed by immunohistochemistry (IHC) staining for phosph-STAT3 (pSTAT3) activation. There were three assigned cohorts: tumor identified with activating mutations in JAK1, JAK2, JAK3, STAT3, or STAT5B; patients lacking in these factors but with functional evidence of JAK/STAT activation determined by ≥30% pSTAT3 staining in tumor cells; and patients who did not fit into cohort 1 and 2, or who lacked adequate tissue suitable for IHC stain. MF patients made up 10% (n=2) of cohort 1, 20% (n=3) of cohort 2, and 12% of cohort 3 (n=2). Clinical benefit rate (CBR) was assessed as a combination of complete response, partial response, or stable disease lasting 6 months. Only one MF patient out of seven whose tumor showed pSTAT3 expression in 20% of cells had a PR to ruxolitinib lasting greater than 18 months. Ruxolitinib had greater success in the PTCL subtype with CBR being 53%, 45%, and 13% in cohorts 1, 2, and 3 respectively (54). An *in vitro* study showed that ruxolitinib in combination with reminostat, an HDAC inhibitor, displayed synergistic antitumor effects in CTCL cell lines and indicated that simultaneous ruxolitinib and reminostat *in vitro* was more effective in inhibiting cell proliferation and inducing apoptosis compared to monotherapy (55). Future studies may shed more insight on the efficacy of ruxolitinib, alone or in combination with other treatments, in the setting of MF/SS.

### Upadacitinib

Upadatitinib is a small molecule JAK inhibitor that is used in the treatment of rheumatoid arthritis and more recently, atopic dermatitis. Upadacitinib’s mechanism of action involves inhibiting JAK1, JAK2, JAK3, and TYK2 enzymes, which are essential components of the JAK-STAT pathway. By blocking these enzymes, upadacitinib can disrupt the abnormal signaling that sustains malignant T-cell growth and inflammatory processes in MF. Although large scale trials have not taken place for the use of upadacitinib in the treatment of MF/SS, a single case report in 2022 illustrated response in a patient with erythrodermic MF. More extensive clinical studies would be needed to establish the efficacy and longitudinal outcomes for upadacitinib in the treatment of MF/SS (56).

## Conclusion

There has been some progress in the treatment landscape of MF and SS, emphasized by monoclonal antibodies, immunotherapies, and JAK inhibitors. These novel therapies allow for a more promising future for patients with refractory or advanced disease.

Monoclonal antibodies offer a more tailored approach to therapy by the precise targeting of specific disease mechanisms. Monoclonal antibodies that specifically target molecules involved in CTCL pathogenesis have been shown to have beneficial therapeutic effects. It should be noted that the monoclonal antibody Dupilumb, an IL-4 and IL-13 inhibitor, has been shown to have the opposite effects. Dupilumab has been more widely used in the treatment of atopic dermatitis, and because CTCL mimics atopic dermatitis in its early stages, Dupilumab has therefore been increasingly administered to CTCL patients (57). Dupilumab was presumed to have some therapeutic potential in MF and SS as disease lesions were demonstrated to have high IL-13 receptor expression (58–60). However, a number of case reports have shown that Dupilumab has the opposite effect and is associated with the progression of CTCL (61).

Immunotherapies can improve the body’s own immune mechanisms to provide a more robust response to disease pathogenesis. JAK inhibitors moreover can target key signaling pathways and disrupt the progression of these diseases. It should be noted however that the data on JAK inhibitor efficacy in CTCL maintenance and therapy is sparse. Future studies should explore the potential of this therapeutic target as there is little clinical exploration in the literature.

The ongoing clinical trials that are discussed in this review highlight the recent progression of research and development for these rare malignancies, and offer areas for innovative approaches to management of MF/SS. A more comprehensive understanding of MF/SS pathophysiology remains critical for improvement in therapeutic response and patient outcomes.

## Author contributions

IQ: Writing – original draft. JR: Writing – review and editing. WH: Writing – review and editing. CC: Writing – review and editing.
